# Effects of an Aqueous Extract of *Withania somnifera* on Strength Training Adaptations and Recovery: The STAR Trial

**DOI:** 10.3390/nu10111807

**Published:** 2018-11-20

**Authors:** Tim N. Ziegenfuss, Anurag W. Kedia, Jennifer E. Sandrock, Betsy J. Raub, Chad M. Kerksick, Hector L. Lopez

**Affiliations:** 1 The Center for Applied Health Sciences, 4302 Allen Road, Suite 120, Stow, OH 44224, USA; Awkedia@sbcglobal.net (A.W.K.); jh@appliedhealthsciences.org (J.E.S.); br@appliedhealthsciences.org (B.J.R.); hl@appliedhealthsciences.org (H.L.L.); 2 Exercise and Performance Nutrition Laboratory, School of Health Sciences, Lindenwood University, 209 S. Kingshighway, St. Charles, MO 63301, USA; ckerksick@lindenwood.edu

**Keywords:** Ashwaganhda, Ayurvedic, resistance training, exercise, placebo, strength, DEXA

## Abstract

*Withania somnifera* (Ashwagandha) is an Ayurvedic herb categorized as having “rasayana” (rejuvenator), longevity, and revitalizing properties. Sensoril® is a standardized aqueous extract of the roots and leaves of *Withania somnifera*. **Purpose:** To examine the impact of Sensoril^®^ supplementation on strength training adaptations. **Methods:** Recreationally active men (26.5 ± 6.4 years, 181 ± 6.8 cm, 86.9 ± 12.5 kg, 24.5 ± 6.6% fat) were randomized in a double-blind fashion to placebo (PLA, *n* = 19) or 500 mg/d Sensoril^®^ (S500, *n* = 19). Body composition (DEXA), muscular strength, power, and endurance, 7.5 km cycling time trial, and clinical blood chemistries were measured at baseline and after 12 weeks of supplementation and training. Subjects were required to maintain their normal dietary habits and to follow a specific, progressive overload resistance-training program (4-day/week, upper body/lower body split). 2 × 2 mixed factorial ANOVA was used for analysis and statistical significance was set *a priori* at *p* ≤ 0.05. **Results:** Gains in 1-RM squat (S500: +19.1 ± 13.0 kg vs. PLA +10.0 ± 6.2 kg, *p* = 0.009) and bench press (S500: +12.8 ± 8.2 kg vs. PLA: +8.0 ± 6.0 kg, *p* = 0.048) were significantly greater in S500. Changes in DEXA-derived android/gynoid ratio (S500: +0.0 ± 0.14 vs. PLA: +0.09 ± 0.1, *p* = 0.03) also favored S500. No other between-group differences were found for body composition, visual analog scales for recovery and affect, or systemic hemodynamics, however, only the S500 group experienced statistically significant improvements in average squat power, peak bench press power, 7.5 km time trial performance, and perceived recovery scores. Clinical chemistry analysis indicated a slight polycythemia effect in PLA, with no other statistical or clinically relevant changes being noted. **Conclusions:** A 500 mg dose of an aqueous extract of Ashwagandha improves upper and lower-body strength, supports a favorable distribution of body mass, and was well tolerated clinically in recreationally active men over a 12-week resistance training and supplementation period.

## 1. Introduction

*Withania somnifera* (ashwagandha) is an Ayurvedic herb belonging to the Solanaceae family. Previous reports have categorized *Withania somnifera* as having “rasayana” (rejuvenator), longevity, and revitalizing properties [1,2,3], but these reports have yet to be fully substantiated using well-controlled, scientific investigations. Ashwagandha has previously been studied in therapeutic areas surrounding cognitive, mood, psychomotor, joint health [2], antioxidation [3], and anti-inflammation. While a firm understanding of many mechanisms of action is not yet present, ashwagandha contains bioactive compounds, including alkaloids (withanine, withasomnin), lactones (withanolides), and glycosides (sitoindosides) that may account for these purported physiological effects [1]. Ashwaganda has also been recognized as having “adaptogenic” properties, which may support a favorable response to the physical and mental stressors of a high-intensity exercise program [1]. Human clinical and animal data in dosages, ranging from 250 to 1000 mg/day, suggest that there is a wide range of physiological effects that may lead to ergogenic benefits, including but not limited to: anxiolytic [4], analgesic [5], anti-inflammatory, anabolic, cardiopulmonary, and antioxidant effects [6]. However, it is important to note that the composition of different *Withania somnifera* extracts depend upon which type of extraction method is used and what part of the plant is undergoing the extraction technique [7]. Limited research has examined the impact of root extracts or other combinations of the plant for their impact on various outcomes.

Sandhu and colleagues [6] were some of the first researchers to examine if Ashwagandha supplementation exerted any impact on muscular or aerobic performance parameters. Using a placebo-controlled approach, 10 healthy participants were assigned to consume either a placebo or Ashwagandha (500 mg/day) for 10 days, and were assessed for changes in power, balance, and maximal oxygen consumption (VO_2_Max). Within-group changes revealed that barbell velocity, power, and VO_2_Max were all increased after ashwagandha supplementation, but no such change was found to occur in the placebo group. The supplement was well tolerated, with no reported adverse events. A later study by Raut et al. [8] enrolled 18 healthy volunteers who were provided increasing dosages of Ashwagandha for a total of 30 days (10 days consecutively at 750 mg/day, 1000 mg/day and, 1250 mg/day) in a prospective, open-label design. All participants were assessed for adverse events as well as changes in strength, exercise tolerance, and body composition using skinfolds. Ashwagandha supplementation was found to reduce total and low-density lipoprotein (LDL) cholesterol and increase muscle strength. Further, body composition tended to improve during the study. Most recently, Wankhede and colleagues [9] reported data on the impact of ashwagandha supplementation (300 mg of root extract, 2×/day) in 57 young, untrained male participants. All participants completed an 8-week course of resistance training while supplementing daily, and had their strength, body composition, and muscle recovery assessed. In these untrained participants taking a root extract of ashwagandha, muscle strength was significantly increased in both the upper-body and lower-body, changes in muscle size using circumference measurements in the arms and chest were greater than changes seen in the placebo group, and reductions in body fat percentage (measured with bioimpedance) were greater in the ashwagandha supplemented group. Additionally, blood markers of damage and recovery, and basal testosterone levels were also slightly improved in the participants taking ashwagandha.

While emerging research has begun to highlight the potential ability of ashwagandha supplementation to improve adaptations seen while resistance training, many studies highlighted above either recruited relatively small numbers of participants or only supplemented for relatively brief periods (<30 days) of time. With the exception of the Wankhede et al. [9] investigation, no research has supplemented with higher dosages while participants completed resistance training for longer than eight weeks. Consequently, more research is needed that span longer supplementation and exercise training protocols in additional study populations at dosages used in previous studies than that supplemented with higher dosages (≥500 mg/day). Furthermore, limited research has explored the impact of an aqueous extract of ashwagandha of both roots and leaves of the native plant. Sensoril^®^ is a standardized aqueous extract of the roots and leaves of *Withania somnifera*, and it contains glycosides, Withaferin-A, and oligosaccharides as major bioactive components. The purpose of this study was to examine the impact of a 500 mg dose of Sensoril^®^ supplementation vs. placebo in recreationally active participants over a 12-week resistance training and supplementation protocol. Primary outcomes were changes in muscle strength and secondary measures were changes in body composition, visual analog scales for recovery and affect, muscle endurance, and power. Based on previous research, it was hypothesized that ashwagandha supplementation would significantly improve muscle strength versus placebo.

## 2. Materials and Methods

### 2.1. Overview of the Study Design

**Figure 1:**
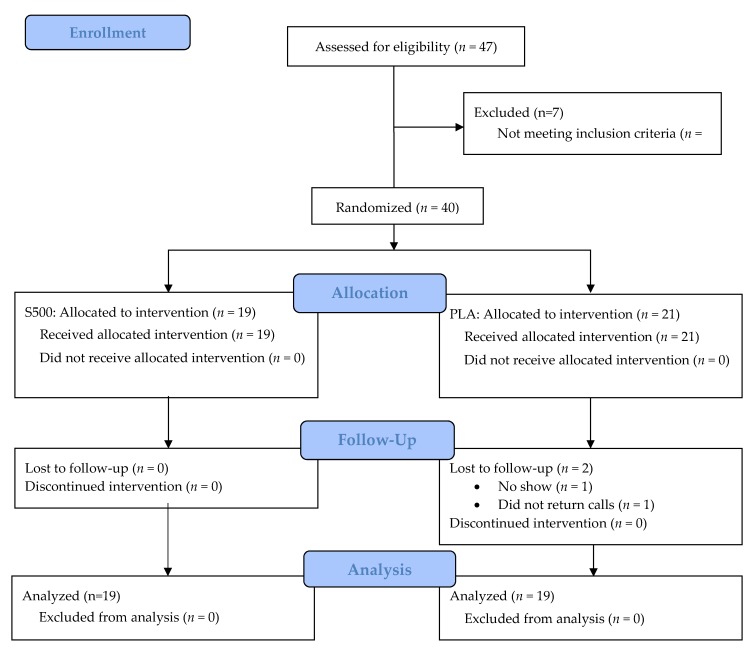
Consolidated Standards of Reporting Trials (CONSORT) diagram.

The study design employed for this protocol was a randomized, double-blind, placebo-controlled investigation that spanned 12 weeks. Each participant completed four study visits (Table 1). The first visit was for screening purposes, and consisted of signing an IRB-approved consent form (IntegReview, Austin, TX, USA; Protocol number: NATSEN-STAR-001-2017; approval date: 19 July 2017) that was in accordance with the Declaration of Helsinki, completing a medical history, recording dietary information, and assessing routine blood work (comprehensive metabolic panel (CMP), complete blood count (CBC), lipid panel). During the second visit, participants were familiarized with the testing protocols used in the study. To evaluate clinical safety, participants had hemodynamic, complete blood counts, comprehensive metabolic, and lipid panels completed after 0 (visit 3) and 12 weeks (visit 4) of supplementation and resistance training. Body composition, dietary habits, upper-body and lower-body strength, power, and endurance, 7.5 km cycling time trial, and various recovery and visual analog scales to assess fatigue/energy, mood, quality of training, and motivation to exercise, were all assessed after 0 and 12 weeks of supplementation. Supplements were consumed once daily in the morning with cold tap water. Prior to all study visits, participants were asked to replicate their previous 24 h dietary intake, abstain from exercise for 48 h and fast for ten hours. Figure 1 provides a CONSORT (Consolidated Standards of Reporting Trials) flow diagram of the study.

### 2.2. Study Participants

Male study participants (*n* = 40) between the ages of 18–45 years (26.3 ± 6.7 years, 1.80 ± 0.07 m, 87.0 ± 12.8 kg) were recruited from the local community in Stow, OH. All participants read and signed an IRB-approved informed consent (IntegReview, Austin, TX, USA; Protocol number: NATSEN-STAR-001-2017; approval date: 19 July 2017) form, that was in accordance with the Declaration of Helsinki, prior to their participation in the study. Upon a review of health/medical history documents and physical exam by a study physician, all study participants were determined to be apparently healthy, recreationally active (training no more than 2–3 days/week for 6–12 months). After screening, all participants had body mass index values between 20–35 kg/m^2^, were normotensive (≤140/≤90 mmHg) and had a normal resting heart rate (≤90 beats/min). Each participant verbally denied using any dietary supplement or finished product (i.e., a pre-workout supplement) that is considered to be ergogenic (i.e., creatine, HMB, beta-alanine, beetroot, phosphates, etc.) in the four weeks prior to screening, as well as during study. Use of a standard strength multivitamin was allowed. 

### 2.3. Participant Demographics

Standing height was determined using a wall-mounted stadiometer with each study participants in socks with heels together. Body weight was measured using a Seca 767^TM^ Medical Scale (Hamburg, Germany, Deutschland). Resting heart rate and blood pressure were measured in duplicate using an automated blood pressure cuff (Omron HEM-780).

### 2.4. Venous Blood Collection

Venous blood samples were collected using standard phlebotomy techniques after 0 and 12 weeks of supplementation and resistance training. Whole blood samples were collected into K_2_-Ethylenediaminetetraacetic acid (EDTA) treated Vacutainer tubes and upon collection were slowly inverted 10 consecutive times prior to immediate refrigeration. Serum samples were collected in serum separator tubes and allowed to clot for 30 min at room temperature prior to being centrifuged (Horizon mini E Centrifuge, Drucker Diagnostics, Port Matilda, PA, USA) for 15 min at 3200 rpm (1600× *g*). All blood samples were analyzed for clinical chemistry analysis (glucose, blood urea nitrogen (BUN), creatinine, aspartate aminotransaminase (AST), alanine aminotransaminase (ALT), creatine kinase, lactate dehydrogenase, total bilirubin, alkaline phosphatase (ALP), triglycerides (TG), total cholesterol (TC), low -density lipoprotein (LDL), very low-density lipoprotein (VLDL), high-density lipoprotein (HDL), uric acid, sodium, potassium, total protein, albumin, globulin, iron, complete blood cells, and platelet count) using automated clinical chemistry analyzers (LabCorp, Dublin, OH, USA). All samples from the same day were batch-analyzed with test-retest reliabilities commonly reported using internal quality control data from clinical laboratories and associated automated analyzers within a range of 3–5% [10].

### 2.5. Body Composition

Lean mass, fat mass, percentage of fat, and android/gynoid ratio were determined by dual-energy x-ray absorptiometry (DEXA; General Electric Lunar DPX Pro) after 0 and 12 weeks of supplementation and resistance training. All DEXA scans were performed by the same technician and analyzed by the manufacturer’s software (enCORE version 13.31, General Electric Healthcare Lunar, Madison, WI, USA); reliability assessments using our device and protocol have been published previously [11]. Briefly, subjects were positioned in the scanner according to standard procedures, and remained motionless for approximately 15 min during scanning. DEXA segments for the upper and lower limbs and trunk were directed using standard anatomical landmarks. Percent fat was calculated by dividing the fat mass by the total scanned mass. Lean-to-fat mass ratio was computed using a simple ratio between the two values. Using the previously defined scanning regions and anatomical landmarks, the android region was defined in the software as the trunk and torso region, while the gynoid region was demarcated by the hip region. From here, android-to-gynoid ratio was computed from the manufacturer software using the pre-defined regions and segments outlined as part of the analysis procedures. Quality control calibration procedures were performed prior to all scans using a calibration block and procedures provided by the manufacturer. Prior to this study, we determined the test–retest reliability for repeated measurements of lean mass, bone mineral content, and fat mass using this DEXA using intra-class correlation coefficients (ICC); all values were ≥0.98 [11].

### 2.6. Muscular Strength

Using standard NSCA (National Strength and Conditioning Association) protocols [12], one-repetition maximum (1-RM) using the Smith machine bench press and back squat were determined as an indicator of maximal upper body and lower body strength, respectively. All maximal attempts were supervised by trained research personnel. Each repetition of the bench press was considered a good repetition if five points of contact (both feet, hips, shoulders, head) were maintained at all points in time and the bar touched the participant’s chest before fully extending the elbows. Similarly, squat repetitions were supervised and spotted, and each repetition was required to go down to a depth where the bottom of the thigh was parallel to the ground. Each subject first performed a warm-up set of eight repetitions at approximately 50% of the perceived 1-RM followed, by a set of three repetitions at 70% of the perceived 1-RM. Thereafter, the subject performed single lifts at progressively heavier weights until failure. No more than five maximal attempts were completed in one testing session. The maximal weight achieved for both the bench press and back squat exercise using these procedures was considered as their 1 RM. Three minutes of rest were given between each maximal attempt, and four minutes rest was given after 1-RM determination before beginning the next assessment. The reliability of these strength testing procedures has been previously determined (ICC, r = 0.994, unpublished data).

### 2.7. Muscular Power

Upper and lower-body power were assessed through the completion of a Smith machine bench press (at 65% of each subject’s 1 RM), and a body weight jump squat while connected a TENDO power analyzer. Previous studies have incorporated the use of a TENDO into their study design [13], and Stock and colleagues [14] have published data to indicate that it is a reliable means of assessment. Reliability in our hands for jump squats (ICC, r = 0.926) and jump throws (ICC, r = 0.921) has been previously determined (unpublished data). The unit consists of a position transducer that measures the rate of linear displacement, providing velocity and acceleration in addition to power production. During the bench press exercise, the TENDO unit was attached to the end of the Smith machine bar. Subjects laid flat on their backs on a bench with their feet on the ground and hands on the bar in a pronated grip. Grip width was standardized for all subjects and reproduced during follow-up testing. Subjects lowered the bar (1–2 s eccentric action) until it lightly touched the chest slightly above the nipple line, and then explosively launched the bar vertically upwards. During the back squat exercise, participants had a TENDO tethered to their waist using a belt before completing three countermovement jumps. For each jump, subjects were required to bend their elbows and place their hands on their hips. Each jump was recorded for peak power and average power. The average of all three collected values for each variable was calculated and used for statistical analysis. Approximately 60–90 s of rest was given between repetitions, and three minutes rest was provided after the completion of the third jump.

### 2.8. Muscular Endurance

To assess upper-body muscular endurance, approximately 65% of the participant’s 1-RM was then loaded onto the bar and each participant was instructed to complete as many repetitions as possible. Each subject completed a total of three sets, interspersed with 60 s of rest. A repetition was only counted if a full range of motion was attained, and once a participant began the set they could not rest for any longer than two seconds at any point throughout the set. The total number of repetitions completed were used as a measure of upper body muscular endurance. Following the third set, a three minute rest period was given before completing the next assessment. Using identical procedures, participants then had their 1-RM determined on the back-squat exercise before resting for four minutes and completing the muscular endurance protocol mentioned above (i.e., 3 sets × as many reps as possible), using the back squat exercise to assess lower-body muscular endurance. The total number of repetitions performed were recorded as their muscular endurance. The reliability of our test procedures was similar to that previously reported [14,15].

### 2.9. Aerobic Endurance

All participants were required to complete a 7.5 km time trial on a computerized cycle ergometer (Velotron, Quarq Technology, Spearfish, SD, USA). Following a 5 min warm up at 75 W, subjects were instructed to complete the course “as quickly as possible”. Participants self-selected their gear ratio and cadence and were blinded to all performance information during the trial, except for elapsed time and distance. All bike measurements (seat height, etc.) and gear selections were recorded and replicated for subsequent follow-up testing. Subjects were permitted to drink water *ad libitum* during the test. The reliability of the Velotron to determine anaerobic power has been previously reported [16].

### 2.10. Visual Analog Scales

Visual analog scales (VAS) were completed by study participants after 0 and 12 weeks of supplementation and resistance training. All VAS were constructed similarly with a 100 mm line anchored by the “Lowest Possible” and “Highest Possible” to assess subjective ratings of mood, desire to workout, willingness to train, optimism, and soreness. The validity and reliability of VAS to assess fatigue and energy have been previously established [17].

### 2.11. Dietary Intake and Control

During the initial screening visit, participants were asked to complete a 24 h dietary recall to assess general habits, food restrictions, and diet composition and intake. Further, 3-day dietary records were completed prior to visits 3 (week 0) and 4 (week 12), and weekly phone calls were used to track dietary compliance. Dietary records were analyzed for average daily energy and macronutrient intake by trained study investigators and NutriBase IX (Clinical Edition) software (CyberSoft, Inc., Phoenix, AZ, USA). Copies of food records were made and provided to each study participant to allow them to standardize their dietary and fluid intake prior to visit 4 [18]. No foods or drinks were allowed in the immediate post-workout period (i.e., 60 min). 

### 2.12. Supplementation

In a randomized, double-blind fashion, participants were assigned to orally ingest either a placebo or a 500-mg dose of a standardized aqueous extract of the roots and leaves of ashwagandha (*Withania somnifera*) (Sensoril^®^, Natreon, Inc., New Brunswick, NJ, USA). Placebo capsules contained rice flour and were of identical size and color. All supplements were blinded by the manufacturer prior to the beginning of the study protocol. The purity and potency of the study product was confirmed by a third party, an independent laboratory. All doses were taken each morning of the study with 12 fluid ounces of cold tap water. Subjects were matched based on training experience, baseline body weight, and strength, prior to being randomized into groups using an online randomization program (www.randomizer.org).

### 2.13. Resistance Training Program

Upon randomization into a supplementation group, study participants were instructed to follow a weekly exercise program. The exercise program was a four day per week periodized resistance-training program designed by a Certified Strength and Conditioning Specialist (CSCS). The workout was designed to train the upper body and lower body two times/week each on a 4-day split (upper body, lower body, upper body, lower body) with gradually increasing volume and intensity based off of the work of Kerksick [19]. The workout consisted of 10–12 exercises, including the following: bench press, lat pulldown, shoulder press, seated row, shoulder shrug, dip, biceps curl, triceps pushdown, leg press, squat, deadlift, lunge, leg curl, leg extension, and calf raise. A simple linear periodization was followed whereby participants trained using three sets of 12–15 RM loads initially and completed the program using 4–6 sets of eight RM loads. For the bench press exercise, a 1-RM % load assignment was used, but for all other loads, loading was used according to repetitions maximums, pre-determined repetition ranges, and following previously instructed loading rules (i.e., 2 × 2 rule). Using this approach, participants were instructed to increase their weight when they could perform two more repetitions than what was prescribed on two consecutive sets. Thus, progression was followed and as strength and endurance improved, training loads were increased to maintain recommended ranges. Rest periods between exercises were 1–3 min and between sets were 1–2 min. Each daily workout was not supervised by study investigators; however, study participants were given a training log to complete for each workout, and each workout was signed off by a training partner or a member of the fitness staff in addition to being monitored during weekly phone calls.

### 2.14. Adverse Events

During weekly phone calls, the frequency and intensity of local and systemic non-serious and serious adverse events (AEs) were recorded by study team members. All reported events were coded using the Medical Dictionary for Regulatory Activities (MedDRA), while the intensity of recorded adverse events were graded using standardized criteria.

### 2.15. Statistical Analysis

All data were entered into two separate Microsoft Excel spreadsheets (i.e., manual double-key data entry), and compared to assure data quality prior to analysis. SPSS 23 (Armonk, NY, USA) was used for all analyses, and data are presented as means ± standard deviations. Normality assumptions were checked on all variables using a one-sample Shapiro–Wilk test. Non-normal distributions were transformed using log_10_. Outliers were checked via visual inspection of studentized calculations on the residuals (threshold value of ±3) of each dependent variable. Independent *t*-tests were used to assess baseline differences. Data were initially analyzed using 2 × 2 mixed factorial ANOVA (group (500 mg, PLA) × time (0 vs. 12 weeks)) with repeated measures on time to determine the presence of any main (time or group) and interaction (group × time) effects. When the sphericity assumption was not met, the Huynh–Feldt correction was applied. The Mean difference of the change scores and the 95% confidence intervals were calculated on the difference between groups using delta values. Within-group effects were compared using a paired samples *t*-test. Effects were considered to be significant at *p* ≤ 0.05, and trends were declared at 0.05 ≤ *p ≤* 0.10.

## 3. Results

Using independent *t*-tests, no baseline differences (*p* > 0.05) were noted for any of the demographic variables (height, body mass, body mass index, body fat %, bench press 1 RM, and squat 1 RM), except age (*p* = 0.04) (Table 2). Based on a review of written logs, the overall compliance to the supplementation and training regimen was 81 and 86%, respectively. Two adverse events occurred in the placebo group (both arthralgia), and three occurred in the supplement group (1 = arthralgia, 1 = myalgia, 1 = abdominal pain). 

### 3.1. Dietary Intake Variables

No significant main effect for time or group × time interaction effects were noted for average calories (Time, *p* = 0.33, Group × Time, *p* = 0.70), carbohydrate (Time, *p* = 0.74, Group × Time, *p* = 0.62), fat (Time, *p* = 0.07, Group × Time, *p* = 0.20), or protein intakes (Time, *p* = 0.62, Group × Time, *p* = 0.29), respectively (Table 3).

### 3.2. Body Composition Variables

A significant group × time interaction effect (*p* = 0.03) was found for the DEXA android/gynoid ratio. Delta values (week 12–week 0) were subsequently computed and assessed using independent *t*-tests, along with 95% confidence intervals on the observed difference between groups. A within-group analysis of the main effect for time revealed no change in S500 while, PLA experienced a significant increase in DEXA-derived android/gynoid ratios. No other statistically significant outcomes were noted for body mass or body composition variables (Table 4).

### 3.3. Visual Analog Scales

Visual analog scales were used to assess perceived recovery, soreness, and several measures of affect. No significant group × time interactions were found for any of the assessed variables (Table 5). Perceived recovery scores significantly improved across time in the S500 group (+14.4%, *p* = 0.003) while no change was noted in the PLA group (+6.7%, *p* = 0.25). Similarly, perceived soreness scores significantly increased in the PLA group (+45.6%, *p* = 0.04), while no change was noted in the S500 group (+18.0%, *p* = 0.37).

### 3.4. Exercise Performance

**Figure 2:**
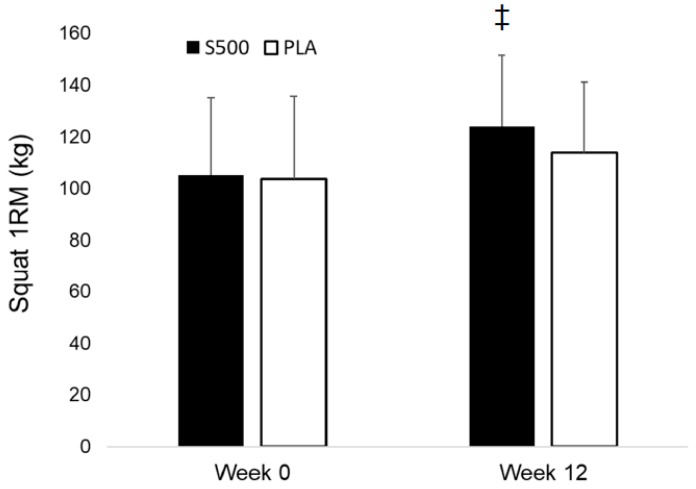
Squat 1 RM in kilograms for S500 and PLA at week 0 and week 12. Solid bars depict S500 data, while white bars depict PLA data. Data is represented as means ± SD. No difference was found between groups at week 0, and is represented as ‡. A significant group × time interaction (*p* = 0.009) with post hoc tests indicating S500 increased squat 1 RM to a greater extent than PLA.

**Figure 3:**
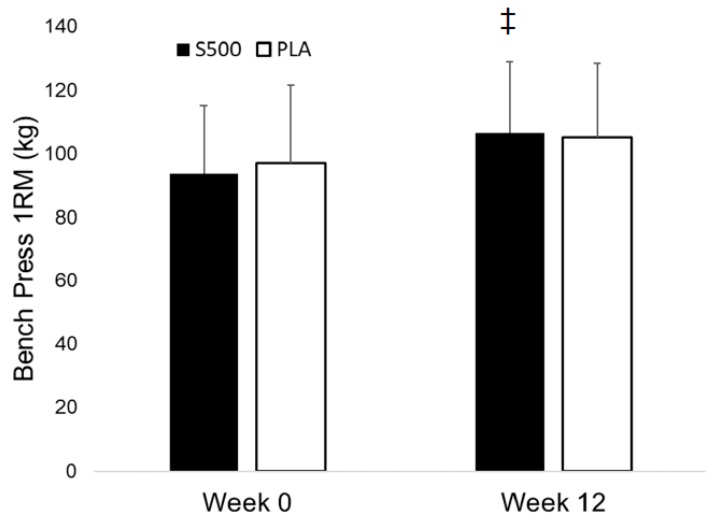
Bench press 1 RM in kilograms for S500 and PLA at week 0 and week 12. Solid bars depict S500 data, while white bars depict PLA data. Data is represented as means ± SD. No difference was found between groups at week 0 and is represented as ‡. A significant group × time interaction (*p* = 0.048) with post hoc tests indicating S500 increased bench press 1 RM to a greater extent than PLA.

Significant group × time interaction effects were found for changes in back squat 1 RM (+18.2% for S500 vs. +9.7% for PLA, *p* = 0.009; 95% CI: (2.4, 15.8 kg)) and bench press 1 RM (+13.7% for S500 vs. +8.2% for PLA, *p* = 0.048; 95% CI: (0.03, 9.52 kg))—see Figure 2 and Figure 3, respectively. Changes in the 7.5 km time trial performance were statistically significant in the S500 group (21% faster, *p* < 0.001), but not in the PLA group (14% faster, *p* = 0.18). Similar changes occurred in average squat power (+4.6%, *p* = 0.007 in S500 vs. +3.1%, *p* = 0.12 in PLA), and peak power during bench press (+11.4%, *p* = 0.007 in S500 vs. +4.7%, *p* = 0.21 for PLA). As shown in Table 6, no other significant group × time interaction effects were found for changes in exercise performance.

### 3.5. Clinical Chemistry

Significant group × time interactions were found for red blood cell count (*p* = 0.04), hemoglobin (*p* = 0.002), hematocrit (*p* = 0.03), total protein (*p* = 0.04), and albumin (*p* = 0.05) (Table 7). For all variables, values increased slightly in the PLA group, but were considered stochastic, and remained within normal clinical limits.

## 4. Discussion

Using a randomized, double-blind, placebo-controlled design, the purpose of the present study was to assess the impact of supplementing with an extract of roots and leaves of the ashwagandha plant (Sensoril**^®^**) on adaptations to strength training in recreationally active, healthy men. The primary findings of this investigation are that significantly greater improvements in both lower-body and upper-body maximal strength occurred when participants were supplementing with a 500 mg dose of ashwagandha as compared to placebo. In addition, ashwagandha supplementation significantly attenuated increases in the android/gynoid ratio as measured by DEXA, and only the ashwagandha group experienced statistically significant improvements in average squat power, peak bench press power, 7.5 km time trial performance, and perceived recovery scores. 

Primary outcomes were defined *a priori* as changes in maximal strength. Based on the existing, yet somewhat limited literature, we hypothesized that ashwagandha supplementation would improve maximal strength to a greater extent when compared to changes seen with placebo. Results from the present study indicated that maximal lower-body and upper-body strength levels, as assessed with a 1-RM maximum squat and bench press, respectively, experienced significantly greater increases after supplementing with a 500 mg dose of ashwagandha in comparison to changes seen in subjects taking a placebo. In addition, average squat power and peak bench press power only increased in the group that supplemented with ashwagandha (S500). Consequently, these results provide additional support that indicate the ability of ashwagandha to improve muscular performance during resistance training. Previously, Sandhu and colleagues [6] reported that ashwagandha supplementation in 40 healthy participants significantly improved barbell velocity, muscular power, and VO_2_Max. Raut et al. [8] used a less rigorous, open-label design in 18 healthy volunteers, and also reported an increase in strength. Furthermore, Wankhede and colleagues [9] supplemented 57 young, but untrained male participants for eight weeks with either 600 mg of ashwagandha (root extract) or placebo while completing a resistance training program, and also reported significantly greater increases in lower-body and upper-body strength. From a mechanistic perspective, the observed improvements in muscular strength while supplementing with ashwagandha are not well understood. Several levels of changes (intramuscular, central nervous, inside the tendon) can occur that can lead to an increase in force production. Unfortunately, the present study and others have not employed approaches to further determine these changes. Future research should employ ultrasound, electromyography, and mechanomyography (MMG) techniques to better understand the neuromuscular reasons for why strength increases have been reported in the ashwagandha literature.

In addition to the performance outcomes, other published reports have highlighted the ability of ashwagandha supplementation to improve various measures of body composition [6,8,9]. For example, Wankehede and colleagues [9] reported significantly greater increases in arm and chest circumference, and greater reductions in body fat percentage (using bioimpedance analysis) in those participants who were supplementing with ashwagandha when compared to a placebo. Results from the present study failed to report any significantly greater improvement in body fat percentage, fat-free mass or fat mass as assessed using a more rigorous assessment tool (e.g., DEXA). A significant group × time interaction (Table 4) was found for the android/gynoid ratio compared to the placebo. The android/gynoid ratio is a metric generated by the DEXA software upon the analysis of each DEXA scan. Pre-defined regions are outlined from each scan, and the DEXA software calculates the total tissue mass found in an android region (trunk) and a gynoid region (hips), and calculates the ratio between these regions. The metric is considered to be a crude marker of visceral fat accumulation. In this respect, an increase in the android/gynoid ratio would be viewed as a less favorable change, due to the known increase in health risks associated with carrying more weight in the torso versus the hips/gluteal region (e.g., insulin resistance, dyslipidemias, etc.) [20,21,22]. In this study, individuals supplementing with ashwagandha experienced no change in this ratio, while participants who supplemented with a placebo experienced a significant increase in the ratio. While this is intriguing, the lack of other associated changes with measured body composition variables makes this finding challenging to interpret. Thus, more research is needed to better understand the potential impact that ashwagandha may have on initiating changes in body composition parameters, particularly any repartitioning of adipose tissue to central regions. Future research should explore assessing changing in various inflammatory biomarkers to identify potential mechanistic considerations for these changes. When viewed collectively, some might question the positive improvements in strength alongside the lack of change seen with lean or fat-free mass levels in the present study. These outcomes, however, have been documented previously. For example, Ahtiainen and colleagues [23] completed a retrospective analysis of 287 individuals between the ages of 19–78 years who had previously performed a resistance training program. As expected, a positive correlation (r = 0.157, *p* = 0.008, *n* = 283) was found between the two responses, but these outcomes varied widely and thus highlight the ability of strength increases to occur without measurable changes in muscle mass. Most importantly, these outcomes clearly point to the need for future studies to more closely investigate the changes seen at the neuromuscular level using techniques such as ultrasound, EMG (electromyography), and MMG (mechanomyography). 

In addition to assessing changes in muscular performance and body composition, we also assessed aerobic exercise performance (via cycling) as well as several self-perception scales of recovery and affect. In line with a previous study that reported significant improvements in VO_2_Max after supplementing with ashwagandha [6], a statistically significant improvement in 7.5 km cycling time trial performance was noted in the ashwagandha-supplemented group (*p* < 0.001) in the present study while no such change was identified in the placebo group (*p* = 0.18). Of note, the group × time interaction effect for this variable did not cross statistically significant thresholds (*p* = 0.48). Additionally, although no significant changes in affect (using various VAS) were found, subjects who supplemented with ashwagandha did demonstrate significant improvements in perceived recovery scores, while no such change was observed in the placebo group. These changes are consistent with previous work, indicating that ashwagandha may help to improve soreness [9], stress [4], and anxiety [1]. Overall, much more work is needed to understand to what extent the type of exercise training, the quality of the subject/athlete, and the exercise stimulus might influence various study outcomes.

This study has several strengths. First, the 12-week supplementation and resistance training program is one of the longest studies to date with any ashwagandha product. Next, participants in this study were already active and participating in various forms of exercise, as opposed to being unaccustomed to physical activity. This is important, as several researchers have identified the impact of training status on adaptations seen from resistance training and other forms of exercise. Finally, this study employed a randomized, double-blind, placebo-controlled design, a gold standard for clinical research. 

We also acknowledge the limitations of this study. While all participants were instructed to follow strict pre-exercise guidelines of rest and fasting, the ability to detect additional changes in performance may have been influenced by study participant’s compliance to these directives. Moreover, while the present study did not overtly seek to maximize hypertrophy adaptations to training, our subjects’ total energy and protein intakes may have undermined their ability to attain more pronounced changes in muscle mass. As an example, relative energy intake was approximately 23.5–25 kcals per kilogram of body mass, and protein intake was ~1.2–1.3 g of protein per kilogram of body mass/day. While potentially adequate, both of these values are lower than recommended amounts to optimize changes in lean mass during resistance exercise training [24,25]. In addition, while weekly contact with subjects was used to gauge compliance with the exercise program, workouts were not strictly supervised by study personnel, and this could have impacted the changes we observed in our study.

Future studies should attempt to more thoroughly control diet, and they include measurements of appetite. In addition, we recommend measuring biomarkers of cardiovascular and metabolic disease risk, including inflammatory cytokines along with utilizing -omic technologies to identify potential changes in novel biomarkers that might assist in potentially establishing an evidence base for this Ayurvedic herb. Finally, methods that permit an examination of motor unit/nervous system activation should be used to help explain the beneficial changes in strength, as noted by us and other authors.

In conclusion, 12 weeks of supplementation with a 500 mg dose of an ashwagandha extract from the plants and leaves (Sensoril^®^), in combination with a progressive, heavy resistance-training program, resulted in significant improvements in maximal lower-body and upper-body strength, and significantly attenuated increases in the android/gynoid ratio. In addition, only the ashwagandha group experienced statistically significant improvements in average squat power, peak bench press power, 7.5 km time trial performance, and perceived recovery scores.

## Figures and Tables

**Table 1:** Overview of research design.

Procedure	Visit 1 Screening	Visit 2 Practice	Visit 3 (Week 0)	Visit 4 (Week 12)
Informed Consent	X			
Inclusion/Exclusion Criteria	X			
Medical History	X			
Physical	X			
Height, weight, BP, HR	X	X	X	X
Safety Screen (CMP, CBC, lipid panel)	X		X	X
Informed Consent	X			
24 h Diet Recall	X			
DEXA–Body Composition			X	X
Visual Analog Scales/PRS		X	X	X
Strength, Endurance, and Power		X	X	X
7.5 km Time Trial		X	X	X
3-Day Diet Records/Analysis			X	X
Protocol Compliance (pill counts and log check)			X	X
Adverse Events Monitoring	X	X	X	X

X = procedure performed, BP = Blood pressure; HR = heart rate; CMP = Comprehensive metabolic panel; CBC = Complete blood counts; DEXA = Dual-energy X-ray absorptiometry; PRS = Perceived recovery status.

**Table 2:** Study participant demographics and dietary intake.

	S500 (*n* = 19)	PLA (*n* = 19)	Significance
Age (years)	24.4 ± 4.2	28.6 ± 7.6	0.04
Height (m)	181 ± 6	180 ± 7	0.68
Body Mass (kg)	85.5 ± 10.5	88.3 ± 14.4	0.50
Body Mass Index (kg/m^2^)	26.2 ± 3.4	27.2 ± 3.9	0.38
Systolic Blood Pressure (mmHg)	124.7 ± 9.7	125.8 ± 8.4	0.71
Diastolic Blood Pressure (mmHg)	71.4 ± 7.7	74.0 ± 8.6	0.33
Resting HR (beats/min)	68.4 ± 9.0	67.5 ± 8.8	0.77
Squat 1 RM (kg)	105.0 ± 30.2	103.8 ± 27.4	0.90
Bench Press 1 RM (kg)	93.7 ± 21.3	97.0 ± 22.4	0.64
DEXA Body Fat (%)	23.8 ± 6.4	25.1 ± 7.0	0.56

S500 = 500 mg daily dose of ashwagandha; PLA = placebo.

**Table 3:** Dietary intake data.

					Between-Group Comparison		
Variables	*n*	Baseline (Week 0)	Post (Week 12)	Within *p*-Value	Mean Difference	95% CI	*p*-Value
Calorie Intake (kcals/day)							
S500 PLA	19 19	2027 ± 568 1956 ± 808	2087 ± 512 2093 ± 1171	0.54 0.45	−77 ± 200	(−483, 329)	0.70
Carbohydrate Intake (g/day)							
S500 PLA	19 19	219 ± 84 214 ± 125	221 ± 78 204 ± 107	0.88 0.62	12.1 ± 24	(−36.4, 60.7)	0.62
Protein Intake (g/day)							
S500 PLA	19 19	104 ± 38 115 ± 39	113 ± 42 112 ± 51	0.32 0.67	11.8 ± 11.1	(−10.6, 34.2)	0.29
Fat Intake (g/day)							
S500 PLA	19 19	78 ± 29 73 ± 32	82 ± 28 94 ± 79	0.88 0.34	−17.1 ± 13.1	(−43.7, 9.4)	0.20

S500 = 500 mg daily dose of ashwagandha; PLA = placebo. 95% CI = 95% confidence interval of the difference between S500 and PLA. Within *p*-value = Factorial ANOVA with repeated measures on time. Between-group *p*-values were calculated using independent *t*-tests using the delta value (week 12–week 0).

**Table 4:** Body composition.

					Between-Group Comparison		
Variables	*n*	Baseline (Week 0)	Post (Week 12)	Within *p*-Value	Mean Difference	95% CI	*p*-Value
Percent Body Fat (%)							
S500 PLA	19 19	23.8 ± 6.4 25.1 ± 7.0	23.2 ± 6.1 25.3 ± 6.5	0.25 0.49	−0.80 ± 0.57	(−1.96, 0.36)	0.17
DXA Lean Mass (kg)							
S500 PLA	19 19	61.9 ± 7.6 62.9 ± 8.4	62.3 ± 7.6 62.9 ± 8.4	0.43 0.91	3.95 ± 5.6	(−7.41, 1.53)	0.49
DXA Fat Mass (kg)							
S500 PLA	19 19	19.7 ± 7.1 21.8 ± 8.6	19.1 ± 6.3 22.0 ± 8.6	0.19 0.57	−8.9 ± 6.3	(−2.2, 3.8)	0.17
DXA Lean/Fat Ratio							
S500 PLA	19 19	3.52 ± 1.33 3.30 ± 1.26	3.64 ± 1.39 3.21 ± 1.09	0.24 0.21	0.21 ± 0.12	(−0.03, 0.44)	0.09
DXA Android/Gynoid Ratio							
S500 PLA	19 19	0.992 ± 0.26 1.095 ± 0.29	0.992 ± 0.28 1.185 ± 0.31	0.99 0.001 †	(−0.09 ± 0.04)	(−0.17, −0.01)	0.03 ‡
DXA Visceral Adipose Tissue (kg)							
S500 PLA	19 19	0.43 ± 0.36 1.02 ± 0.98	0.41 ± 0.34 0.99 ± 0.93	0.37 0.53	0.05 ± 49.1	(−99.6, 99.7)	0.99
Body Mass (kg)							
S500 PLA	19 19	85.5 ± 10.5 88.3 ± 14.4	84.9 ± 10.5 87.7 ± 14.3	0.52 0.58	0.03 ± 1.35	(−2.71, 2.77)	0.98

S500 = 500 mg daily dose of ashwagandha; PLA = placebo. 95% CI = 95% confidence interval of the difference between S500 and PLA. Within *p*-value = Factorial ANOVA with repeated measures on time. Between-group *p*-values were calculated using independent *t*-tests using the delta value (week 12–week 0). † indicates significant within-group differences, ‡ indicates significant between group differences.

**Table 5:** Visual analog scales.

					Between-Group Comparison		
Variables	*n*	Baseline (Week 0)	Post (Week 12)	Within *p*-Value	Mean Difference	95% CI	*p*-Value
Perceived Recovery Score							
S500 PLA	19 19	6.58 ± 1.22 7.00 ± 1.37	7.53 ± 1.02 7.47 ± 1.90	0.003 † 0.25	0.47 ± 0.48	(−0.51, 1.45)	0.33
Invigorated							
S500 PLA	19 19	6.58 ± 1.30 6.43 ± 1.64	6.75 ± 1.31 6.78 ± 1.43	0.64 0.31	−0.18 ± 0.49	(−1.18, 0.82)	0.72
Mood							
S500 PLA	19 19	7.22 ± 1.25 6.56 ± 1.67	7.61 ± 1.17 7.24 ± 1.50	0.19 0.08 †	−0.28 ± 0.47	(−1.22, 0.67)	0.55
Desire to Workout							
S500 PLA	19 19	6.46 ± 1.55 6.46 ± 1.56	7.08 ± 1.57 7.05 ± 1.62	0.18 0.13	0.04 ± 0.58	(−1.14, 1.22)	0.95
Willingness to Train							
S500 PLA	19 19	7.41 ± 1.67 7.01 ± 1.84	7.92 ± 1.40 7.59 ± 1.35	0.27 0.07 †	−0.07 ± 0.53	(−1.16, 1.01)	0.89
Optimism							
S500 PLA	19 19	7.37 ± 1.86 7.23 ± 1.98	7.88 ± 2.09 7.79 ± 1.46	0.25 0.09 †	−0.06 ± 0.53	(1.13, 1.02)	0.91
Soreness							
S500 PLA	19 19	3.33 ± 1.87 2.63 ± 2.19	3.93 ± 2.47 3.83 ± 2.37	0.37 0.04 †	−0.61 ± 0.83	(−2.30, 1.09)	0.47

S500 = 500 mg daily dose of ashwagandha; PLA = placebo. 95% CI = 95% confidence interval of the difference between S500 and PLA. Within *p*-value = Factorial ANOVA with repeated measures on time. Between-group *p*-values were calculated using independent *t*-tests using the delta value (week 12–week 0). † indicates significant within-group differences.

**Table 6:** Exercise performance.

					Between-Group Comparison		
Variables	N	Baseline (Week 0)	Post (Week 12)	Within *p*-Value	Mean Difference	95% CI	*p*-Value
7.5 km Time Trial (seconds)							
S500 PLA	19 19	1241 ± 268 1189 ± 389	977 ± 119 1020 ± 189	<0.001 † 0.18	−95.5 ± 133	(−366, 174)	0.48
Average Power All Sets Squats (Watts)							
S500 PLA	19 19	1443 ± 206 1442 ± 240	1510 ± 220 1486 ± 227	0.007 † 0.12	19.2 ± 41.1	(−64.1, 102.4)	0.52
Peak Power All Sets Squats (Watts)							
S500 PLA	19 19	2424 ± 347 2444 ± 271	2628 ± 197 2580 ± 217	<0.001 † 0.01 †	68.5 ± 67.4	(−68.1, 205.1)	0.32
Squat 1-RM (kg)							
S500 PLA	19 19	105.0 ± 30.2 103.8 ± 27.4	124.1 ± 32.0 113.9 ± 27.2	<0.001 † <0.001 †	9.1 ± 3.3	(2.4, 15.8)	0.009 ‡
Squat Repetitions All Sets (repetitions)							
S500 PLA	19 19	29.8 ± 11.7 32.7 ± 12.0	36.6 ± 10.0 44.4 ± 19.5	0.01 † 0.002 †	−4.9 ± 4.2	(−13.4, 3.5)	0.24
Bench Press 1-RM (kg)							
S500 PLA	19 19	93.7 ± 21.3 97.0 ± 22.4	106.5 ± 24.7 105.0 ± 23.3	<0.001 † <0.001 †	4.78 ± 2.34	(0.03, 9.52)	0.048 ‡
Average Bench Press Power All Sets (Watts)							
S500 PLA	19 19	379 ± 101 396 ± 127	423 ± 107 423 ± 111	0.005 † 0.04	17.1 ± 18.8	(−21.1, 55.3)	0.37
Peak Power Bench Press All Sets (Watts)							
S500 PLA	19 19	492 ± 131 529 ± 154	548 ± 133 554 ± 156	0.007 † 0.21	30.4 ± 26.3	(−23.0, 83.8)	0.26
Bench Press Repetitions Completed All Sets							
S500 PLA	19 19	29.8 ± 5.3 30.8 ± 8.4	38.2 ± 9.6 36.8 ± 12.4	<0.001 † 0.03 †	2.4 ± 3.1	(−3.95, 8.69)	0.45

S500 = 500 mg daily dose of ashwagandha; PLA = placebo. 95% CI = 95% confidence interval of the difference between S500 and PLA. Within *p*-value = Factorial ANOVA with repeated measures on time. Between-group *p*-values were calculated using independent *t*-tests using the delta value (week 12–week 0). † indicates significant within-group differences, ‡ indicates significant between group differences.

**Table 7:** Serum and whole blood clinical markers of safety.

					Between-Group Comparison		
Variables	N	Baseline (Week 0)	Post (Week 12)	Within *p*-Value	Mean Difference	95% CI	*p*-Value
White blood cell count (cells/μL)							
S500 PLA	19 19	5.88 ± 1.36 5.54 ± 1.50	6.48 ± 1.40 5.68 ± 1.83	0.14 0.68	0.46 ± 0.51	(−0.56, 1.49)	0.37
Red blood cell count (cells/μL)							
S500 PLA	19 19	5.08 ± 0.29 5.07 ± 0.36	5.08 ± 0.24 5.21 ± 0.42	0.97 0.01 †	−0.15 ± 0.07	(−0.29, −0.01)	0.04 ‡
Hemoglobin (g/dL)							
S500 PLA	19 19	15.5 ± 0.24 15.4 ± 0.90	15.2 ± 0.53 15.8 ± 0.94	0.03 † 0.02 †	−0.64 ± 0.19	(−1.03, −0.26)	0.002 ‡
Hematocrit (%)							
S500 PLA	19 19	43.5 ± 2.42 43.4 ± 2.33	43.5 ± 1.75 44.7 ± 2.87	0.85 0.01 †	−1.37 ± 0.62	(−2.63, −0.11)	0.03 ‡
Glucose (g/dL)							
S500 PLA	19 19	87.9 ± 8.4 89.5 ± 6.3	90.8 ± 4.7 90.4 ± 5.7	0.18 0.63	2.11 ± 2.69	(−3.35, 7.56)	0.44
Blood Urea Nitrogen (mg/dL)							
S500 PLA	19 19	14.5 ± 3.55 15.1 ± 3.85	14.3 ± 3.0 14.7 ± 3.1	0.72 0.60	0.16 ± 0.91	(−1.69, 2.00)	0.86
Creatinine (mg/dL)							
S500 PLA	19 19	1.01 ± 0.15 0.98 ± 0.13	1.08 ± 0.14 1.02 ± 0.13	0.03 † 0.11	0.02 ± 0.04	(−0.05, 0.10)	0.56
Blood Urea Nitrogen/Creatinine Ratio							
S500 PLA	19 19	14.5 ± 3.2 15.8 ± 5.3	13.5 ± 2.9 14.8 ± 4.1	0.19 0.24	−0.05 ± 1.13	(−2.34, 2.23)	0.96
Sodium (mEq/L)							
S500 PLA	19 19	141.6 ± 1.39 141.2 ± 1.64	141.1 ± 1.24 140.5 ± 1.78	0.29 0.13	0.21 ± 0.61	(−1.02, 1.45)	0.73
Potassium (mEq/L)							
S500 PLA	19 19	4.44 ± 0.32 4.40 ± 0.22	4.33 ± 0.26 4.34 ± 0.21	0.10 0.21	−0.04 ± 0.08	(−0.20, 0.12)	0.59
Chloride (mEq/L)							
S500 PLA	19 19	101.1 ± 1.99 100.9 ± 1.70	101.2 ± 1.99 100.6 ± 1.77	0.77 0.48	0.47 ± 0.70	(−0.94, 1.89)	0.50
Carbon Dioxide (mEq/L)							
S500 PLA	19 19	24.7 ± 1.77 24.1 ± 1.78	24.3 ± 1.73 23.2 ± 2.41	0.34 0.18	0.47 ± 0.77	(−1.09, 2.03)	0.54
Protein (g/dL)							
S500 PLA	19 19	7.36 ± 0.43 7.15 ± 0.28	7.20 ± 0.40 7.31 ± 0.33	0.16 0.12	−0.32 ± 0.14	(−0.61, −0.02)	0.04
Albumin (g/dL)							
S500 PLA	19 19	4.80 ± 0.30 4.74 ± 0.19	4.67 ± 0.25 4.78 ± 0.20	0.04 † 0.53	−0.16 ± 0.08	(−0.33, 0.00)	0.05
Globulin (g/dL)							
S500 PLA	19 19	2.56 ± 0.26 2.41 ± 0.28	2.53 ± 0.25 2.53 ± 0.34	0.72 0.23	−0.15 ± 0.13	(−0.41, 0.11)	0.24
Albumin/Globulin Ratio							
S500 PLA	19 19	1.89 ± 0.22 2.00 ± 0.29	1.86 ± 0.21 1.93 ± 0.28	0.60 0.42	0.03 ± 0.11	(−0.19, 0.25)	0.77
Bilirubin (mg/dL)							
S500 PLA	19 19	0.58 ± 0.32 0.71 ± 0.45	0.59 ± 0.28 0.74 ± 0.41	0.88 0.52	−0.03 ± 0.09	(−0.21, 0.16)	0.77
Alkaline Phosphatase (U/L)							
S500 PLA	19 19	66.4 ± 14.4 73.9 ± 21.4	64.2 ± 11.5 74.3 ± 19.9	0.46 0.85	−2.58 ± 3.5	(−9.68, 4.52)	0.47
Aspartate Aminotransferase (U/L)							
S500 PLA	19 19	24.3 ± 9.2 24.8 ± 7.8	22.8 ± 8.2 25.9 ± 8.1	0.24 0.61	−2.68 ± 2.56	(−7.88, 2.51)	0.30
Alanine Aminotransferase (U/L)							
S500 PLA	19 19	20.6 ± 9.1 24.8 ± 7.8	22.8 ± 8.2 25.9 ± 8.1	0.88 0.24	−2.37 ± 2.40	(−7.23, 2.50)	0.33
Total Cholesterol (mg/dL)							
S500 PLA	19 19	157.2 ± 31.9 181.3 ± 44.9	155.2 ± 28.6 184.3 ± 43.5	0.51 0.49	−5.0 ± 5.2	(−15.5, 5.5)	0.34
Triglycerides (mg/dL)							
S500 PLA	19 19	72.7 ± 21.7 130.4 ± 114.8	80.5 ± 35.6 116.5 ± 66.	0.36 0.38	21.7 ± 17.5	(−13.8, 57.2)	0.22
High Density Lipoprotein Cholesterol (mg/dL)							
S500 PLA	19 19	53.3 ± 10.8 47.7 ± 7.2	51.4 ± 11.5 47.9 ± 7.7	0.23 0.86	−2.16 ± 1.95	(−6.11, 1.80)	0.28
Very Low Density Lipoprotein Cholesterol (mg/dL)							
S500 PLA	19 19	14.4 ± 4.5 21.8 ± 14.2	16.1 ± 7.0 23.3 ± 13.4	0.33 0.94	1.74 ± 2.23	(−2.78, 6.27)	0.44
Low Density Lipoprotein Cholesterol (mg/dL)							
S500 PLA	19 19	89.4 ± 30.5 107.1 ± 34.3	87.8 ± 25.3 113.2 ± 36.6	0.59 0.33	−6.19 ± 5.34	(−17.0, 4.6)	0.25

S500 = 500 mg daily dose of ashwagandha; PLA = placebo. 95% CI = 95% confidence interval of the difference between S500 and PLA. Within *p*-value = Factorial ANOVA with repeated measures on time. Between-group *p*-values were calculated using independent *t*-tests using the delta value (week 12–week 0). † indicates significant within-group differences, ‡ indicates significant between group differences.

## Availability of Data and Materials

The data and materials for this manuscript are not scheduled to be made publicly available due to the proprietary nature of the investigated materials. Contractually, the data are owned by Natreon, Inc.
